# Active Enhancement of Slow Light Based on Plasmon-Induced Transparency with Gain Materials

**DOI:** 10.3390/ma11060941

**Published:** 2018-06-03

**Authors:** Zhaojian Zhang, Junbo Yang, Xin He, Yunxin Han, Jingjing Zhang, Jie Huang, Dingbo Chen, Siyu Xu

**Affiliations:** 1 College of Liberal Arts and Sciences, National University of Defense Technology, Changsha 410073, China; 376824388@sjtu.edu.cn (Z.Z.); zhangjingjing13@nudt.edu.cn (J.Z.); jhuang_nudt@163.com (J.H.); c_dingbo@163.com (D.C.); 13149600390@163.com (S.X.); 2 Center of Material Science, National University of Defense Technology, Changsha 410073, China; xinhestudy@163.com (X.H.); hanyx15@163.com (Y.H.)

**Keywords:** plasmon-induced transparency, metal-dielectric-metal, gain material

## Abstract

As a plasmonic analogue of electromagnetically induced transparency (EIT), plasmon-induced transparency (PIT) has drawn more attention due to its potential of realizing on-chip sensing, slow light and nonlinear effect enhancement. However, the performance of a plasmonic system is always limited by the metal ohmic loss. Here, we numerically report a PIT system with gain materials based on plasmonic metal-insulator-metal waveguide. The corresponding phenomenon can be theoretically analyzed by coupled mode theory (CMT). After filling gain material into a disk cavity, the system intrinsic loss can be compensated by external pump beam, and the PIT can be greatly fueled to achieve a dramatic enhancement of slow light performance. Finally, a double-channel enhanced slow light is introduced by adding a second gain disk cavity. This work paves way for a potential new high-performance slow light device, which can have significant applications for high-compact plasmonic circuits and optical communication.

## 1. Introduction

Electromagnetically induced transparency (EIT), which arises from the quantum destructive interference between two distinct excitation channels in a three-level atomic system [1], has potential in slow light [2] and nonlinear optical response enhancement [3] due to its excellent ability to modulate dispersion. However, EIT requires strict experimental conditions such as low temperature and stable pumping [1], which makes it hard to be applied in practical optical systems, especially on-chip devices. As an analogue of EIT in a plasmonic system, plasmon-induced transparency (PIT) has drawn more attention. PIT is attributed to the destructive interference between plasmonic radiative mode and subradiative mode [4], which can be realized on metamaterials [4] and nanoscale plasmonic circuits [5]. Nowadays, PIT can be found extensively in nano applications for sensing [6,7,8], filtering [9,10], and slow light [11,12,13,14,15,16].

Although PIT can be tuned by changing geometric parameters, it is essential to propose an active modulation of PIT in practical applications, and thus various optical materials are utilized to achieve this target. Superconductor and polymer are used to obtain temperature modulation of transparent window position [9,17], and nonlinear Kerr material can realize ultrafast all-optical control [13,18]. Utilizing 2D material graphene, both transparent window position and intensity can be dynamically adjusted by external electric gating voltage [19,20,21]. Although tunable PIT ensures good control of delay time for slow light applications, such delay time always has a threshold because the dispersion modulation of PIT is restricted by the intrinsic loss (ohmic loss) of metal. In particular, the delay time can only reach approximately 1 picosecond (ps) at most [14] in the plasmonic metal-dielectric-metal (MDM) system. Some studies have found that PIT can be dramatically enhanced by applying gain material to overcome the loss, and such gain-assisted plasmonic devices exhibit brilliant performance as a spaser (nanolaser) [22,23], sensor [24,25], and buffer [26,27]. However, few works have focused on gain-assisted PIT in MDM system. Compared to dielectric waveguide, plasmonic MDM waveguide can confine optical modes in deep subwavelength to break the diffraction limit, which is a solution for the next generation of highly integrated on-chip circuits [28]. Pumped active plasmonic waveguides may suffer from the self-heating effect [29], but the problem can be solved by a heat sink system [30].

In this paper, PIT is investigated numerically based on plasmonic MDM waveguide system side-coupled with a stub and disk resonator. The corresponding phenomenon can be theoretically analyzed by coupled mode theory (CMT). After filling gain material into a disk cavity, PIT can be greatly boosted by external pump beam to achieve a dramatic enhancement of slow light performance. Finally, a double-channel enhanced slow light is introduced by adding a second gain disk cavity. This work paves way for a potential new high-performance slow light device, which can have significant applications in high-compact plasmonic circuits and optical communication.

## 2. Materials and Methods

**Figure 1:**
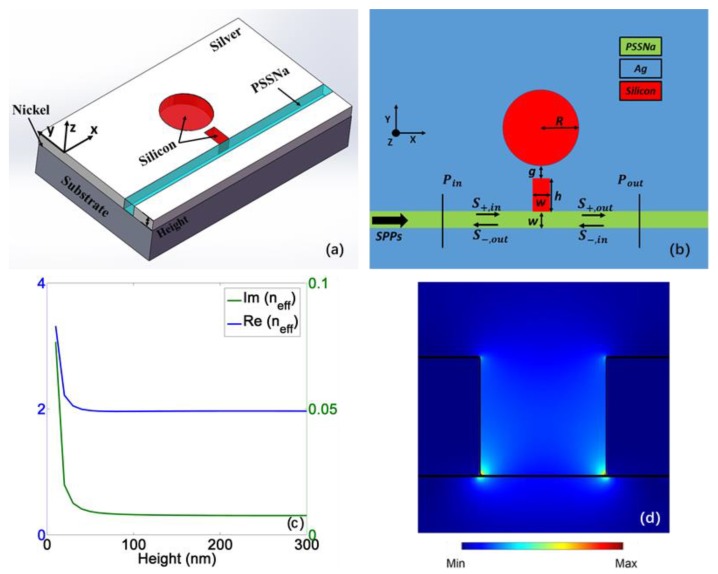
(**a**) 3D scheme of the plasmonic metal-dielectric-metal (MDM) waveguide system; (**b**) 2D scheme of this system from the view of *z*-axis. The geometry parameters are *w* = 100 nm, *h* = 205 nm, *g* = 19 nm, *R* = 141 nm; (**c**) The relationship between the effective refractive index (*n_eef_*) of surface plasmon waves (SPWs) and the height at a wavelength of 1310 nm; (**d**) The distribution $\left|P_{x}\right|$ of the fundamental mode at 1310 nm when height is 100 nm.

In Figure 1a, the plasmonic MDM waveguide system is presented in 3D image, and the 2D scheme from the view of *z*-axis is shown in Figure 1b; the detailed structural geometric parameters are given in the caption. Here, nickel (Ni) is utilized to promote adhesion between metal and substrate [31]. For the height of the MDM waveguide, the relationship between the surface plasmon waves (SPWs) effective refractive index ($n_{eff}$) and the height at the wavelength of 1310 nm (which is the main wavelength discussed in this paper) is given in Figure 1c, where the width of waveguide is fixed at 100 nm. When the height rises at start, both real and imaginary part of the $n_{eff}$ will drop owing to the reduction of the modal power fraction at interfaces [32]. The decrease in $Im\left(n_{eff}\right)$ indicates less propagation loss and, consequently, longer propagation length. However, after reaching a certain height, $n_{eff}$ will keep stable. Therefore, the height can be selected as 100 nm according to the outcome. The distribution $\left|P_{x}\right|$ of the fundamental mode at 1310 nm when height is 100 nm is shown in Figure 1d.

To describe the permittivity of Ag, the Drude model is utilized as follows [6]:

(1) $$\varepsilon_{m}=\varepsilon_{\infty }-\frac{\omega_{p}^{2}}{\omega (\omega +i\gamma )}$$

where *ε*_∞_ is the dielectric permittivity of the infinite frequency, $\omega_{p}$ refers to the bulk frequency for plasma, *γ* is the damping frequency for electron oscillation, and *ω* gives the incident light angular frequency. The corresponding parameters of Ag are $\varepsilon_{\infty }$ = 3.7, $\omega_{p}$ = 1.38 × $10^{16}$ Hz, and γ = 2.73 × 10^13^ Hz. The stub and disk cavities are both filled with silicon (Si, ε=12.25) [33]. To ensure the stability of the guided light propagation as well as resist the corrosion and oxidation, the waveguide should be filled with dielectric materials. For optical interconnects, optical polymer is a good choice for several reasons [34]. First, polymer has the compatibility with existing manufacturing processes of conventional plasmonic waveguides. Second, it can be easily integrated into existing architectures by the spin coating method. Third—and most importantly—it can withstand the high-temperature environment of a pumped system. Additionally, it is low-cost. Here, sodium p-styrenesulfonate (PSSNa) homopolymer with a refractive index of 1.395 [31] is selected. Compared with other conventional optical polymers such as polymethyl methacrylate (PMMA) [35], PSSNa has a lower refractive index and therefore suffers from less propagation loss.

In MDM waveguide, only transverse-magnetic (TM) mode can exist [36]. Compared to incident wavelength, the width of the waveguide is much smaller, so there is only fundamental TM mode. The dispersion relation of this fundamental mode is described as follows [36]:

$$\frac{\varepsilon_{i}p}{\varepsilon_{m}k}=\frac{1-e^{kw}}{1+e^{kw}}$$

(2) $$k=k_{0}\sqrt{{\left(\frac{\beta_{spp}}{k_{0}}\right)}^{2}-\varepsilon_{i}},\;p=k_{0}\sqrt{{\left(\frac{\beta_{spp}}{k_{0}}\right)}^{2}-\varepsilon_{m}}$$

$$\beta_{spp}=n_{eff}k_{0}=n_{eff}\frac{2\pi }{\lambda }$$

Here, *w* refers to the width of the waveguide, *λ* is incident light wavelength in vacuum, *ε_i_* and *ε_m_* give the dielectric and metal permittivity, *β_spp_* is propagation constant of SPWs, and $k_{0}=2\pi /\lambda$ is the wave number. The 2D finite-difference time-domain (FDTD) solution with mesh grid size 2 nm is utilized to simulate this device with the boundary condition of stabilized perfectly matched layers (PML) to maintain convergence. To collect the incident and transmitted power, two monitors are put at *P_in_* and $P_{out}$, respectively, as shown in Figure 1b. The transmission spectrum of power is calculated as *T* = *P_out_*/*P_in_*.

**Figure 2:**
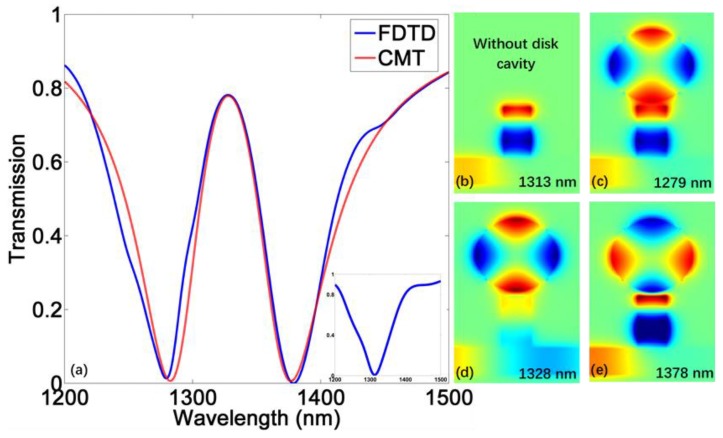
(**a**) The transmission spectrum from finite-difference time-domain (FDTD) and coupled mode theory (CMT). The inset is the transmission spectrum without disk cavity; (**b**–**e**) The corresponding *H_z_* distribution. For plasmon-induced transparency (PIT), the central transparent wavelength is 1328 nm, the valley values are at 1279 nm and 1378 nm.

The transmission spectrum of this structure from FDTD is depicted in Figure 2a, which possesses a PIT profile. The spectrum without disk cavity is given in the inset, showing a band-stop spectral characteristic. The corresponding distribution of $H_{z}$ are given in Figure 2b–e. In a plasmonic waveguide system, PIT results from the destructive interference between radiative mode (directly excited mode) and subradiative mode (indirectly excited mode). Here, both stub and disk cavities can act as resonators. The stub cavity can be seen as a Fabry–Perot (F–P) resonator, with the F–P mode (FPM) corresponding to the radiative mode. The disk resonator possesses a whispering gallery mode (WGM) that serves as the subradiative mode. The resonance conditions are respectively given as follows [37]:

(3) FPM:mλ=2h⋅Re(neff),m=1,2…WGM:kdHn(1)′(keR)Hn(1)(keR)=keJn′(kdR)Jn(kdR),n=1,2…

where *R* is the disk radius, $k_{d}$ and $k_{e}$ are the wave vectors in the disk resonator and metal, respectively, $H_{n}^{\left(1\right)}$ and $H_{n}^{{\left(1\right)}^{\prime }}$ are the first kind Hankle function with order *n* and its derivative, respectively, and *J_n_* and $J_{n}^{\prime }$ are the first kind Bessel function with order *n* and its derivative, respectively. *m* and *n* refer to the mode number which is an integer. According to the field distribution in Figure 2, *m* = 1 and *n* = 2. The generating mechanism of PIT can be theoretically analyzed by CMT [15]. As demonstrated in Figure 1b, $S_{\pm ,in}$ and $S_{\pm ,out}$ represent amplitudes of input and output SPWs, respectively; the subscript ± means two directions of wave propagation. The decay rates of stub and disk resonators are indicated as *α* and *β*, respectively, which arises from the resonator intrinsic loss. The coupling coefficient between the bus waveguide and stub resonator is given as γ, and δ is the coupling coefficient between the two resonators. According to temporal CMT, the field amplitudes *a* and *b* that correspond to FPM and WGM, respectively, can be described as follows [15]:

(4) $$\begin{array}{l} \frac{da}{dt}=(j\omega_{0}-\alpha -\gamma )a+j\sqrt{\gamma }(S_{+in}+S_{-in})+j\delta b\\ \frac{db}{dt}=(j\omega_{0}-\beta )b+j\delta a \end{array}$$

where $\omega_{0}$ is the common resonant frequency of two resonators, which is also the central frequency of the transparent window, and *j* is an imaginary unit. According to power conservation and time reversal symmetry, the input and output wave amplitudes have a relationship as follows:

(5) $$\begin{array}{l} S_{+,out}=S_{+,in}+j\sqrt{\gamma }a\\ S_{-,out}=S_{-,in}+j\sqrt{\gamma }a \end{array}$$

Here, $S_{-,in}$ = 0 because SPWs is input from the left. Based on Equations (4) and (5), the transmission spectrum can be calculated as:

(6) $$T(\omega )={\left|\frac{S_{+,out}}{S_{+,in}}\right|}^{2}={\left|1-2\gamma \frac{2j(\omega -\omega_{0})+2\beta }{{\left[2j(\omega -\omega_{0})+\alpha +\beta +\gamma \right]}^{2}+{\left(2\delta \right)}^{2}-{\left(\alpha -\beta +\gamma \right)}^{2}}\right|}^{2}$$

The transmission data from CMT is also plotted in Figure 2a, showing that the theoretical curve is in great agreement with the simulated data.

## 3. The Slow Light Performance

**Figure 3:**
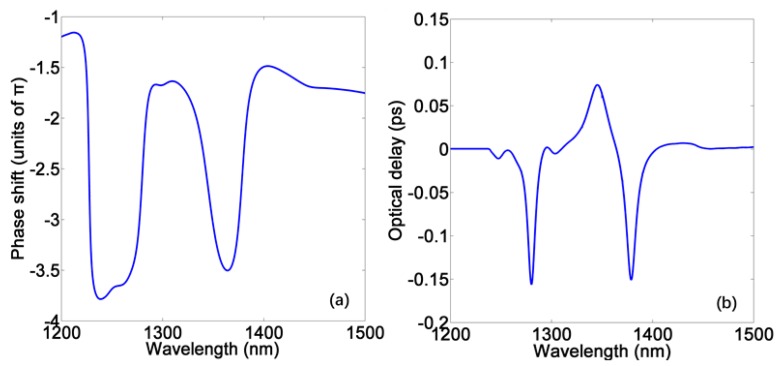
(**a**) The spectrum of phase shift in no-gain PIT system; (**b**) The spectrum of delay time in no-gain PIT system.

Slow light effect is an indispensable part of PIT applications. The spectrum profile of PIT indicates that there is an extreme modification of the dispersion properties within the transparent window. In an anomalous dispersion regime, a group velocity large than *c* (the speed of light in vacuum) leads to a superluminal pulse propagation, and such fast light can be utilized for gravitational wave detection and rotation sensing [38]. In a normal dispersion regime, the group velocity can be slower than *c* and a subluminal pulse propagation can be obtained, which is called slow light [39]. Here we only focus on the slow light effect. The slow light performance can be assessed by optical delay time $\tau_{g}$ and group index $n_{g}$ described as follows [12]:

(7) $$\begin{array}{l} \tau_{g}=\frac{d\psi (\omega )}{d\omega }\\ n_{g}=\frac{c}{v_{g}}=\frac{c}{D}\tau_{g} \end{array}$$

where *ψ*(*ω*) stands for the transmission phase shift from the light source to the monitor, *c* is the light speed, *v_g_* is the group velocity in the plasmonic waveguide, and *D* is the length of this system. Since the length of the different devices are not the same, it is more accurate to evaluate the slow light performance by optical delay time. Figure 3a,b show the phase shift and delay time of the device in Figure 1b; the positive and negative delay time represent slow and fast light effect, respectively. We can find a slope of phase shift within the transparent window, producing a delay time of approximately 0.074 ps around the PIT peak. Such values are low because this PIT profile is not very sharp, consequently producing a relatively gentle dispersion. It is known that a wider gap between two resonators can reduce the corresponding coupling coefficient to produce a narrower PIT peak with a steeper slope [14,15], which can lead to a stronger dispersion. However, it will also bring more intrinsic loss of metal, causing lower transmission of slow light that will make the device inefficient.

## 4. Slow Light Enhanced by Gain Material

**Figure 4:**
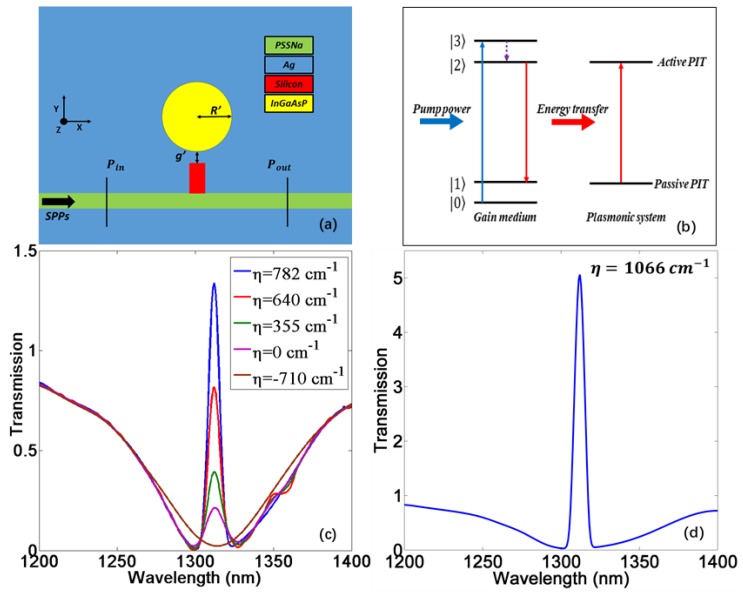
(**a**) 2D scheme of gain-assisted PIT system from the view of *z*-axis. The geometry parameters are *g*′ = 41 nm, *R*′ = 149 nm; (**b**) Schematic of the energy transfer from a pumped four-level gain medium to the PIT resonance in plasmonic system; (**c**,**d**) The transmission spectrum with different gain coefficients.

To compensate for the intrinsic loss in the plasmonic system, optical gain materials such as quantum dots and dye molecules have been utilized [40,41]. Here, we use the semiconductor InGaAsP $\left(\varepsilon =11.38-i\varepsilon_{I}\right)$ [33] as an active media to fill the disk cavity as shown in Figure 4a. The interaction between pump beam and gain medium can be described by a four-level quantum system shown in Figure 4b [24]. Inside the gain media, the pump photons can excite an electronic transition from the ground state |0〉 to the highest excited state |3〉, then come to a metastable state |2〉 via a fast nonradiative transition. Next, the signal light will act as the trigger to make gain material return to a lower level |1〉 by radiating photons with same frequency as signal light. Consequently, pump energy is transferred to the plasmonic system, providing the compensation for the system intrinsic loss. One of the schemes to introduce pump beam is from the input of waveguide. However, the most common pump wavelength for InGaAsP is 980 nm [42,43], which cannot be coupled into gain material as there is no resonant behavior inside such disk cavity at 980 nm. Therefore, pump beam should be directly injected on the gain material from the top (*z*-axis) of this device [44].

In this simulation, the imaginary part of the active medium permittivity can represent the loss or gain. *ε_I_* will be −0.1 without the pump beam, and the positive imaginary part of permittivity indicates the case of loss. When increasing the pump power, $\varepsilon_{I}$ will rise and eventually become positive, and the medium will show the gain effect with a negative imaginary part of dielectric constant. Here, the gain coefficient $\eta =-\left(2\pi /\lambda \right)Im\sqrt{11.38-i\varepsilon_{I}}$ is utilized to describe the gain level [22].

Figure 4c,d demonstrate the PIT transmission spectrum with different gain coefficients of which central transparent wavelength is at conventional telecommunication wavelength of 1310 nm. When $\eta =-710{\;\mathrm{cm}}^{-1}$ ($\varepsilon_{I}=-0.1)$, which corresponds to no pumping [33], there is no transparent peak. Because the system is lossy under this condition, the electric field in the disk attenuates quickly so the WGM (subradiative mode) cannot be formed. As the pump power increases, the intrinsic loss of system will be compensated gradually. Therefore, the suppressed PIT will be released, leading to the rise of a transparent peak. When the peak value reaches the unity, it indicates that the metallic intrinsic loss is fully compensated by the gain. After that, the surplus gain power will be efficiently delivered to the PIT resonance via energy matching as the gain level continues increasing, leading to the amplification of the SPWs. Therefore, the transparent peak will exceed the unity and keep rising as shown in Figure 4d. [24]. Such phenomenon can be applied to realize nanolasers [23].

**Figure 5:**
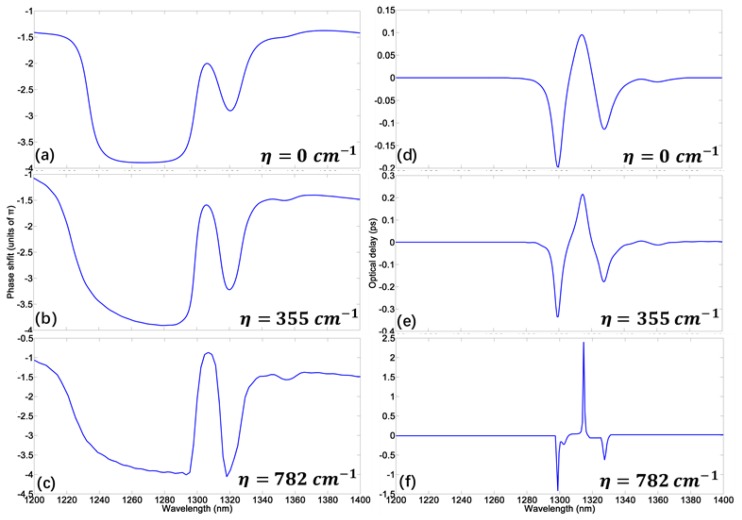
(**a**–**f**) The phase shift and delay time corresponding to three different gain levels.

**Figure 6:**
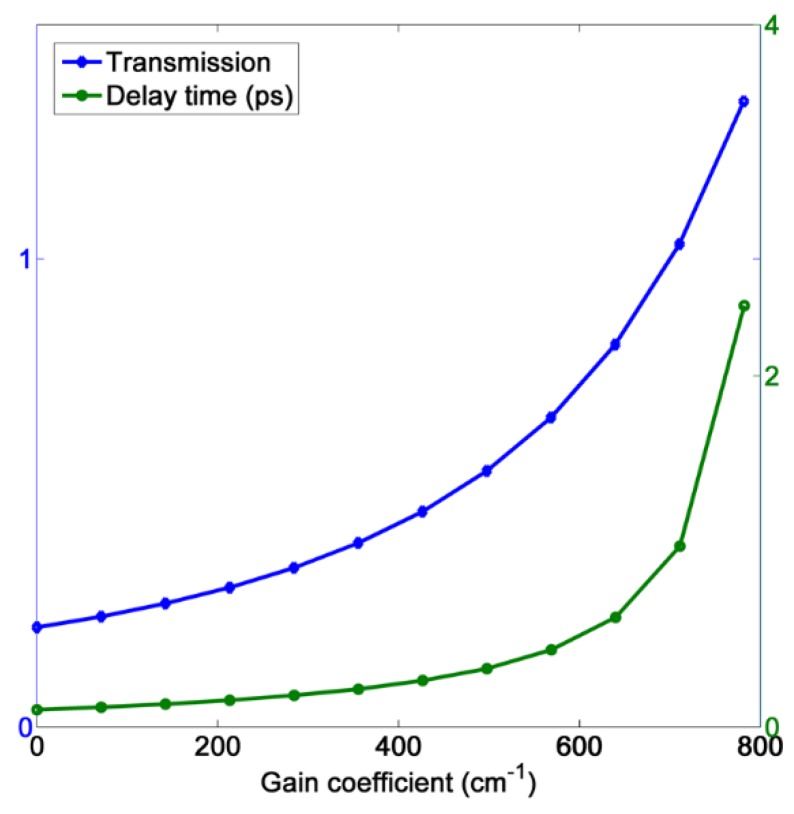
The relationship between transmission/delay time and gain coefficients at 1310 nm.

For a slow light system, however, the excessive gain may risk damage to the detector due to the ultrahigh output intensity. Therefore, we only focus on the slow light effect of PIT with peak value near unity. The phase shift and delay time corresponding to three different gain levels are exhibited in Figure 5a–f. These show that as the gain level rises, the transmission peak will increase and the delay time is dramatically enhanced simultaneously. Figure 6 displays the relationship between transmission/delay time and gain coefficient at 1310 nm. When $\eta =782\;{\mathrm{cm}}^{-1}$, the delay time can be improved up to 2.4 ps in this plasmonic MDM system with a high transmission at 1310 nm, which is greater than any previous similar passive systems [12,13,14,15,16]. At the same time, the transmission of transparent peak is 1.34, which is only a little higher than the unity. Such excellent slow light performance can be attributed to two reasons. First, the gain compensation for the ohmic loss allows for a wider gap between two resonators and consequently produces a narrow transparent peak with considerable amplitude. Second, the high gain level can boost the peak value, making the peak slope steeper. Such extremely sharp PIT profile will lead to a strong group velocity dispersion at the transparent range. It has to be mentioned that the highest gain level above is still within the range of current semiconductor gain materials [45,46].

## 5. Gain-Assisted Slow Light with Double Channels

**Figure 7:**
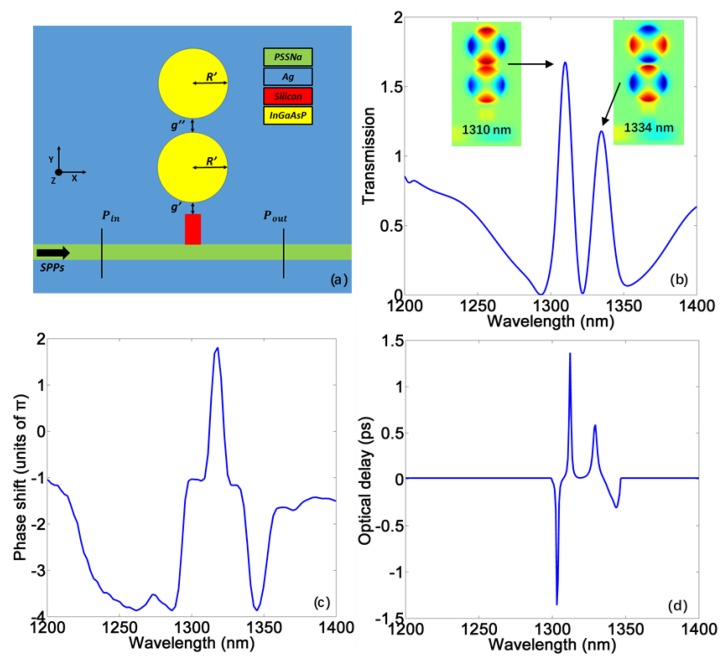
(**a**) 2D scheme of the gain-assisted double PIT system from the view of *z*-axis. The geometry parameters are *g*′ = 28 nm, *g*″ = 36 nm, *R*′ = 149 nm; (**b**) The transmission spectrum of double-disk system under the gain level $\eta =782\;{\mathrm{cm}}^{-1}$. The insets are field distributions corresponding to the two central transparent wavelengths; (**c**) The phase shift spectrum of corresponding double PIT; (**d**) The delay time of corresponding double PIT.

By introducing the second gain-filled disk cavity, double boosted PIT can be achieved as shown in Figure 7a,b. The field distributions corresponding to the two transparent windows are given in the inset of Figure 7b. From this, we can see that the combination of different WGMs can produce two kinds of subradiative modes, leading to the double PIT. Under the gain level *η* = 7820 cm^−1^, both transparent peaks can be fueled by the active gain, bringing double-channel enhanced slow light performance as shown in Figure 7c,d. At two different channels, the delay time can reach 1.37 ps and 0.59 ps, respectively.

## 6. Conclusions

In summary, we report a gain-assisted plasmonic MDM system with a superior slow light performance based on PIT effect. Both the transmission and optical delay of slow light can be dramatically enhanced by the gain power. Finally, a double-channel, enhanced slow light can be achieved by introducing another disk cavity. After applying the gain material, the device performance can be enhanced and we can also realize the active control of the PIT peak and delay time. To propose such a structure, Ag film can be deposited on the Ni surface by thermal evaporation method [31], and the bus waveguide and cavities can be etched on the Ag layer by focused ion beam (FIB) [9]. Such a system also has potential applications for bio-sensing, nanolaser and optical switching.
